# Investigating a Detection Method for Viruses and Pathogens Using a Dual-Microcantilever Sensor

**DOI:** 10.3390/mi15091117

**Published:** 2024-08-31

**Authors:** Luca Banchelli, Georgi Todorov, Vladimir Stavrov, Borislav Ganev, Todor Todorov

**Affiliations:** 1 Department of Theory of Mechanisms and Machines, Faculty of Industrial Technology, Technical University of Sofia, 1797 Sofia, Bulgaria; esoitaly@gmail.com; 2 Department of Manufacturing Technology and Systems, Faculty of Industrial Technology, Technical University of Sofia, 1797 Sofia, Bulgaria; gdt@tu-sofia.bg; 3 AMG Technology Ltd., Microelectronica Industrial Zone, 2140 Botevgrad, Bulgaria; vs@amg-t.com; 4 Department of Electronics, Faculty of Electronic Engineering and Technologies, Technical University of Sofia, 1797 Sofia, Bulgaria

**Keywords:** microcantilever, piezoresistor, vibration, virus detection, SARS-CoV-2

## Abstract

Piezoresistive microcantilever sensors for the detection of viruses, pathogens, and trace chemical gasses, with appropriate measurement and signal processing methods, can be a powerful instrument with high speed and sensitivity, with in situ and real-time capabilities. This paper discusses a novel method for mass sensing on the order of a few femtograms, using a dual-microcantilever piezoresistive sensor with a vibrating common base. The two microcantilevers have controllably shifted natural frequencies with only one of them being active. Two active piezoresistors are located on the surfaces of each of the two flexures, which are specifically connected in a Wheatstone bridge with two more equivalent passive resistors located on the sensor base. A dedicated experimental system measures the voltages of the two half-bridges and, after determining their amplitude–frequency responses, finds the modulus of their differences. The modified amplitude–frequency response possesses a cusp point which is a function of the natural frequencies of the microcantilevers. The signal processing theory is derived, and experiments are carried out on the temperature variation in the natural frequency of the active microcantilever. Theoretical and experimental data of the temperature–frequency influence and equivalent mass with the same impact are obtained. The results confirm the sensor’s applicability for the detection of ultra-small objects, including early diagnosis and prediction in microbiology, for example, for the presence of SARS-CoV-2 virus, other viruses, and pathogens. The versatile nature of the method makes it applicable to other fields such as medicine, chemistry, and ecology.

## 1. Introduction

The detection of chemical substances, viruses, and pathogens with masses in the femtogram range when performed by microcantilever sensors is distinguished by their low power consumption, high sensitivity, and fast response. An undoubted advantage of these sensors is that, under appropriate conditions, the detection of the chemical or the diagnosis of the presence of a particular virus can be made at an early stage, in situ in real time, even before the contamination process or harmful chemical saturation has occurred.

Single microcantilever sensors have already been applied in various fields, for example, to detect C-reactive protein (CRP) [1] or to determine optical bias for single-nucleotide mismatch recognition [2]. The simplified design of these sensors is a prerequisite for their lower cost and low power consumption and creates opportunities for the application of simple electronic circuits and signal processing software. On the other hand, compared to dual-microcantilever sensors, they have lower temperature compensation and lower sensitivity and reliability.

In dual-microcantilever sensors, one of the most popular detection methods is based on comparing the natural frequencies of a passive and active microcantilever. Tian X. et al. demonstrated dual-microcantilever sensors containing a microcantilever for hydrogen sulfide gas detection, which compared with metal oxide gas sensors have ultra-low power and high sensitivity in certain cases [3]. The ratio of amplitudes in a coupled dual-microcantilever beam has been used as a sensing factor by achieving noise suppression, smaller damping forces, and the larger mass difference of the microcantilevers [4]. A dual-microcantilever sensor with high sensitivity and trace hydrogen sulfide gas detection capabilities with positive and negative frequency shifts has been demonstrated in [5]. In some studies on the application of piezoelectric polymer two-microcantilever sensors, it has been shown that higher sensitivity is achieved when operating with the second order of natural frequencies [6].

The operating principles of microcantilever piezoresistive sensors are mainly divided into static and dynamic. In the former, the signal is produced by the static deformation of the microcantilever, which affects the parameters of an electrical circuit through the piezoresistive effect [7,8]. In dynamic methods, the microcantilever is forced to vibrate, for example, by photo- or electro-thermal effects [9,10], by the vibratory actuation of the substrate [11,12], or by other actuation characteristics of microelectromechanical systems (MEMS), such as piezoelectric [13], capacitive [14], or magnetic actuation [15].

In terms of the application of microcantilever sensors, more versatile applications are envisaged beyond those in microbiology or chemistry. Genesensors obtained by modifying the surface of a microcantilever with applications in biology, chemistry, pharmaceutics, and environmental monitoring have been systematically studied in terms of immobilization processes, complementary hybridization, and signal extraction and processing [16].

The theory for the study of microcantilever sensors includes both lumped and distributed dynamic models. It is well known that distributed parameter models give a clearer picture of the behavior of the objects under study, but due to their complexity, they pose some theoretical difficulties, for example, in describing resonant modes. A dynamic distributed parameter model of a cantilever with base excitation and tip mass was presented by To C. in [17], and studies of forced vibrations near the resonant mode were modeled by Repetto et al. in [18]. Alternatively, lumped parameter models find applications in solving a variety of problems, such as those related to antistiction problems [19] or for static and modal analysis [20].

The aim of this paper is to elucidate and experimentally validate the theoretical basis of a new method for the detection of viruses, pathogens, and chemical gasses with masses in the order of several femtograms, based on the measurement of the frequency of a cusp point in the amplitude–frequency response of two microcantilevers. The research presented here summarizes some of the work of several research projects and is based on a recent patent-applied method [21]. In a publication by Banchelli et al. [22], the patented method was investigated with respect to its robustness and sustainability.

## 2. Sensor Description and Problem Statement Formulation

**Figure 1:**
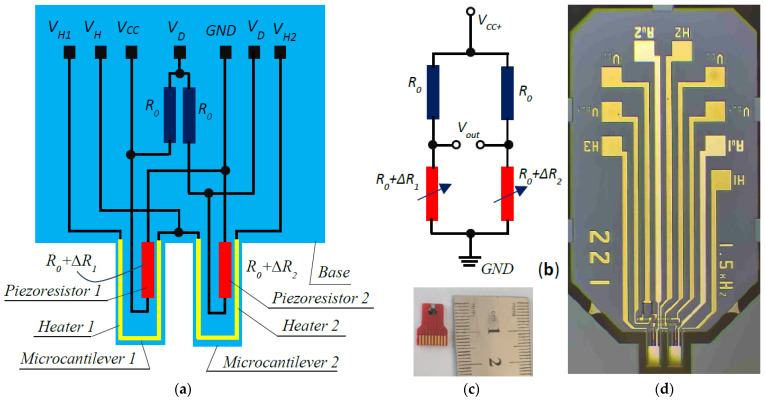
Schematic overview and topology of dual-cantilever microsensor: (**a**) electromechanical schematic; (**b**) Wheatstone bridge circuit; (**c**) photo of sensor; (**d**) close-up view of sensor topology.

The sensor consists of two silicon microcantilevers with a common base. One piezoresistor with a resistance $R_{0}$ is located on the surface of each of the microcantilevers and two equivalent resistors are added at the base. Thin-film aluminum U-shaped heaters were fabricated on the two microcantilevers, as shown in Figure 1a. The piezoresistors and passive resistors were connected in a Wheatstone bridge, as shown in Figure 1b. Through the two passive resistors, temperature compensation is achieved in the output signal of the Wheatstone bridge. Figure 1c reveals the appearance of the sensor. The close-up of the sensor shown in Figure 1d shows that thin-film gold-coated pads are patterned on the two microcantilevers, and each can be selectively activated for detection, while the other performs a passive function. The gold pads are not shown in Figure 1a.

The principle of operation of the sensor is similar in operation to most dual-microcantilever sensors with an active and passive microcantilever but differs in the method of signal detection. The two microcantilevers have close but controllably shifted natural frequencies. The base of the two cantilevers vibrates with a monotonically varying frequency swept in a narrow range around the natural frequencies of the sensor. During the sweep at the frequency from the Wheatstone bridge, a signal is measured that has a point in its amplitude–frequency response curve between the two natural frequencies of the microcantilevers with an amplitude lower than the sensor noise level. The change in frequency of the zero point is sensitive enough to register a change in the mass of one of the cantilevers relative to the other on the order of femtograms. This principle will be elucidated in detail later.

## 3. Load and Dynamic Model of Cantilever Beam with Harmonic Base Excitation

**Figure 2:**
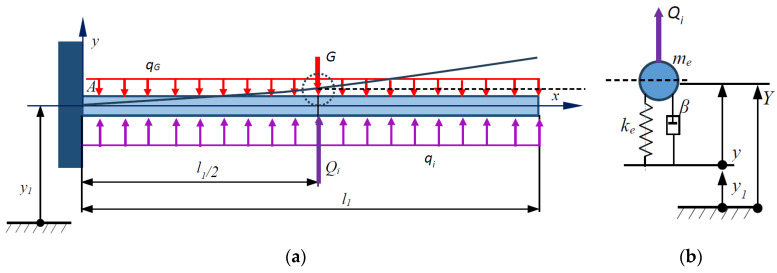
Microcantilever beam with harmonically driven base: (**a**) microcantilever diagram; (**b**) lumped dynamic model of microcantilever beam.

The base of the microcantilevers is assumed to move by a harmonic function $y_{1}$ of the form

(1) $$y_{1}=a\sin\omega t,$$

where a is the amplitude of the excitation function, ω is the circular frequency, and t is the time. The axis x of the absolute coordinate system Axy is assumed to coincide with the neutral longitudinal line of the beam, and the deformation transverse displacements are performed along the axis y (Figure 2a). The dynamic model with lumped microcantilever parameters is depicted in Figure 2b.

The microcantilever has a length $l_{1}$ and a constant rectangular cross section with width $l_{2}$ and height $l_{3}$.

Consider an elementary cantilever volume of length dx, located at an arbitrary distance x from the fixation point A. On this volume due to the acceleration of the base,

(2) $$\frac{d^{2}y_{1}}{dt}=-a\omega^{2}\sin\omega t$$

an inertial elementary force

(3) $$d\phi =-\left(-a\omega^{2}\sin\omega t\right)dm$$

acts with the help of the elemental mass dm, which is calculated by

(4) $$dm=\rho l_{2}l_{3}dx,$$

where ρ is the density of silicon, the material from which the microcantilever is made.

The inertial elementary force is distributed along the length of the microcantilever with the longitudinally distributed load

(5) $$q_{i}=\frac{d\phi }{dx}=\rho al_{2}l_{3}\omega^{2}\sin\omega t.$$

In addition, the regularly distributed weight $q_{G}$ acts on the cantilever:

(6) $$q_{G}=\frac{dG}{dx}=-\rho al_{2}l_{3}g,$$

where

(7) $$dG=-g\rho l_{2}l_{3}dx$$

is the weight of the elementary volume, and g is the gravity acceleration.

The two longitudinally uniformly distributed loads have equivalent concentrated inertial $Q_{i}$ and weight G forces, which are located in the middle of the cantilever and, respectively, have the form

(8) $$Q_{i}=q_{i}l_{1}=\rho al_{1}l_{2}l_{3}\omega^{2}\sin\omega t=ma\omega^{2}\sin\omega t,$$

(9) $$G=q_{G}l_{1}=-g\rho l_{1}l_{2}l_{3}dx.$$

Since the cantilever vibrates at frequencies as high as 50 kHz, and the amplitude of the vibrations is greater than 1 µm, the acceleration generated by the base motion exceeds the ground acceleration by at least two orders of magnitude, warranting the neglection of the gravitational force. Another reason for neglecting this loading is that it causes deformations of the microcantilever on the order of several angstroms (Å) [23].

The microcantilever loaded as such, also referred to as the original, is fitted with a concentrated model [23], which is a mass point located in the middle of the microcantilever on which the applied inertial force acts (Figure 2b). The mass point moves according to the same law as that of the midpoint on the neutral line of the cantilever. The concentrated model has an effective mass $m_{e}$ and an effective stiffness $k_{e}$, obtained under the condition of equality of the model and original energies.

The motion of the concentrated mass is described by the differential equation

(10) $$m_{e}\frac{d^{2}y}{dt^{2}}+b\frac{dy}{dt}+k_{e}y=Q_{i}$$

where b is the viscous resistivity coefficient.

After taking (8) into account and dividing by $m_{e}$, (10) is rewritten as

(11) $$\frac{d^{2}y}{dt^{2}}+2\beta \frac{dy}{dt}+\varpi^{2}y=a_{e}\omega^{2}\sin\left(\omega t\right),$$

where

(12) $$\beta =\frac{b}{2m}$$

is the damping coefficient,

(13) $$\varpi =\sqrt{\frac{k}{m}}$$

is the natural frequency of the microcantilever, and

(14) $$a_{e}=\frac{ma}{m_{e}}$$

is the effective amplitude of the forced vibration.

The solution of the Linear Differential Equation (10) is obtained as the sum of the solution of the homogeneous equation and a partial integral of the inhomogeneous equation. The solution of the homogeneous equation is damped and vanishes after a short time. This is a reason to take the solution of (11) as only the forced oscillations of the microcantilever, which, for the case under consideration, have the form

(15) $$y=B\sin\left(\omega t+\psi \right),$$

where the amplitude B of the forced oscillations is

(16) $$B=\frac{a\omega^{2}}{\sqrt{{\left(\omega^{2}-\varpi^{2}\right)}^{2}-4\beta^{2}\omega^{2}}}$$

and the forced vibration phase has the form

(17) $$\psi =\frac{2\omega \beta }{\omega^{2}-\varpi^{2}}.$$

The piezoresistors are formed in the fixed end of the cantilever, where the mechanical stress σ is calculated using the formula

(18) $$\sigma =\frac{M_{y}}{W},$$

where the bending moment $M_{y}$ is determined by the expression

(19) $$M_{y}=\frac{Q_{i}l_{1}}{2}=-\frac{l_{1}^{2}q}{2}$$

The force $Q_{i}$ is substituted according to (8), and the resisting moment $W_{y}$ is calculated using the formula

(20) $$W_{y}=\frac{l_{2}l_{3}^{2}}{6}.$$

The transverse displacement of the static elastic line of the microcantilever under a uniformly linear distributed load is given by

(21) $$w\left(x\right)=\frac{qx^{2}}{24EI}\left(6l_{1}^{2}-4l_{1}x+x\right),$$

where

(22) $$I_{y}=\frac{l_{2}l_{3}^{3}}{12}$$

is the moment of inertia of the cross section with respect to the axis x.

At the midpoint of the beam at $x=\frac{l_{1}}{2}$ and with the help of (21), the deflection $w_{2}$ is found

(23) $$w_{2}=w\left(\frac{l_{1}}{2}\right)=\frac{17l_{1}^{4}q}{384EI}$$

From the above formula, q is expressed and substituted into (19), and after taking into account that $w_{2}\equiv y$, the relationship between the midpoint displacement and the fixed-end mechanical stress is obtained:

(24) $$\sigma =\frac{96El_{3}}{17l_{1}^{2}}y\left(t\right)=\frac{96El_{3}}{17l_{1}^{2}}B\sin(\omega t+\psi ).$$

## 4. Basic Concepts of the Considered Piezoresistor Detection Method

At the ends of piezoresistor 1 of the Wheatstone bridge (Figure 1b), the electrical voltage is obtained

(25) $$u_{1}=\frac{R_{0}+\Delta R_{1}}{2R_{0}+\Delta R_{1}}v_{cc}.$$

Similarly, the end-to-end voltage of piezoresistor 2 is measured:

(26) $$u_{2}=\frac{R_{0}+\Delta R_{2}}{2R_{0}+\Delta R_{2}}v_{cc}.$$

In Formulas (25) and (26), since silicon is an anisotropic material according to [24] and [25], the relative resistance change $\Delta R_{1}$ in the piezoresistor is proportional to the mechanical stress, and for plane (100) and direction [110] the relations for the two piezoresistors, respectively, are

(27) $$\begin{array}{l} \frac{\Delta R_{1}}{R_{0}}=\pi_{l}(1+\nu \pi_{t})\sigma_{1}=\pi_{R}\sigma_{1}\\ \frac{\Delta R_{2}}{R_{0}}=\pi_{l}(1+\nu \pi_{t})\sigma_{2}=\pi_{R}\sigma_{2} \end{array},$$

where in plane (100) for direction [110], $\pi_{l}=\frac{1}{2}\left(\pi_{11}+\pi_{12}+\pi_{44}\right)$ and $\pi_{t}=\frac{1}{3}\left(\pi_{11}+2\pi_{12}-\pi_{44}\right)$, ν is Poisson’s ratio, $\pi_{R}=\pi_{l}(1+\nu \pi_{t})$, and $v_{cc}$ is the supply voltage.

Having considered the formulas in (27), $\sigma_{1}$ and $\sigma_{2}$, assuming a form according to (24) for the electrical voltages of the two piezoresistors, are obtained:

(28) $$\begin{array}{l} u_{1}=\frac{1+\delta_{R}B_{1}\sin(\omega t+\psi_{1})}{2+\delta_{R}B_{1}\sin(\omega t+\psi_{1})}v_{cc},\\ u_{2}=\frac{1+\delta_{R}B_{2}\sin(\omega t+\psi_{2})}{2+\delta_{R}B_{2}\sin(\omega t+\psi_{2})}v_{cc}, \end{array}$$

where

(29) $$\delta_{R}=\frac{96El_{3}\pi_{R}}{17l_{1}^{2}}$$

is called the generalized piezoresistivity coefficient.

The functions in (28) are periodic and at the time points

(30) $$\begin{array}{l} t=\frac{4\pi n+\pi -2\psi_{i}}{2\omega }\\ t=\frac{4\pi n-\pi -2\psi_{i}}{2\omega }\; \end{array}n=1,\;2,\;3\ldots \;i=1,2,$$

these have the following maximum and minimum values, respectively:

(31) $$\begin{array}{l} u_{\max i}=\frac{1+\delta_{R}B_{i}}{2+\delta_{R}B_{i}}v_{cc},\\ u_{\min i}=\frac{1-\delta_{R}B_{i}}{2-\delta_{R}B_{i}}v_{cc}\;\;\;\;\;\;i=1,2. \end{array}$$

It is assumed that the amplitude is the measured peak-to-peak voltage; therefore, the amplitude–frequency response function is represented by

(32) $$V_{Ai}\left(\omega \right)=u_{\max i}-u_{\min i}=\frac{2\delta_{R}B_{i}\left(\omega \right)}{4-\delta_{R}^{2}B_{i}^{2}\left(\omega \right)}v_{cc}\;\;\;\;\;i=1,2.$$

**Figure 3:**
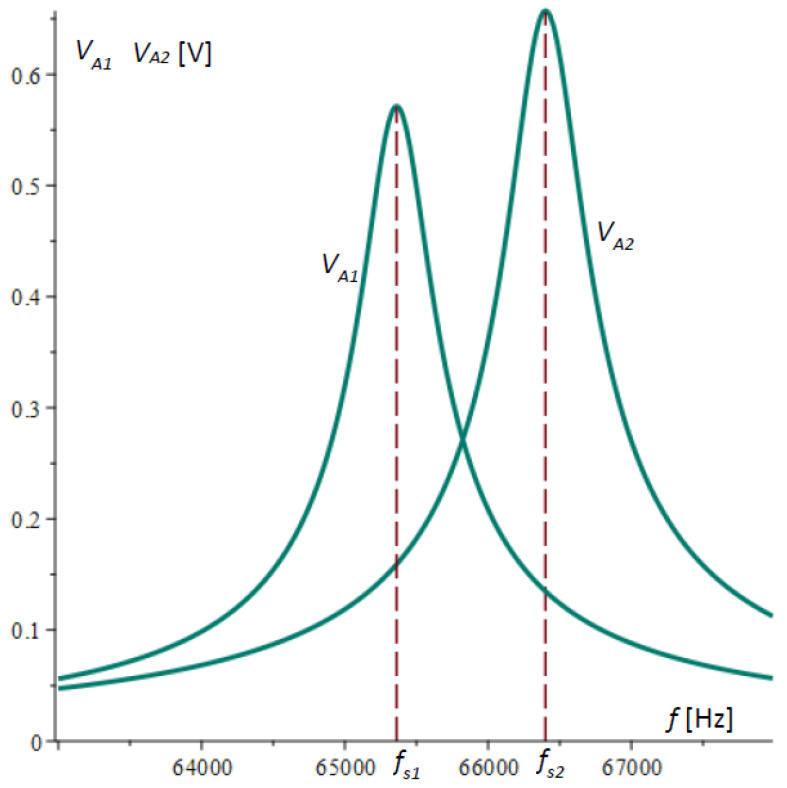
Calculated amplitude–frequency responses of microcantilever 1 $V_{A1}$ [V] and microcantilever 2 $V_{A2}$ [V] as function of frequency f [Hz].

Figure 3 shows the plots of the amplitude–frequency characteristics of microcantilever 1 and microcantilever 2 obtained by Equation (32) and the data in Table 1.

The output voltage of the Wheatstone bridge [29] is calculated using the formula

(33) $$V_{out}=u_{1}-u_{2}=\frac{\left[B_{1}\sin\left(\omega t+\psi_{1}\right)-B_{2}\sin\left(\omega t+\psi_{2}\right)\right]\delta_{r}v_{cc}}{\left[2+\delta_{r}B_{1}\sin\left(\omega t+\psi_{1}\right)\right]\left[2+\delta_{r}B_{2}\sin\left(\omega t+\psi_{2}\right)\right]},$$

which was obtained after taking into account Formula (28) and making simplifications.

For the experimental study of the output voltage amplitude, the difference is measured here:

(34) $$V_{Aout}=V_{A1}-V_{A2}=\frac{2\delta_{R}\left(B_{1}-B_{2}\right)\left(B_{1}B_{2}\delta_{R}^{2}+4\right)}{\left(\delta_{R}^{2}B_{i}^{2}-4\right)\left(\delta_{R}^{2}B_{2}^{2}-4\right)}v_{cc}.$$

**Figure 4:**
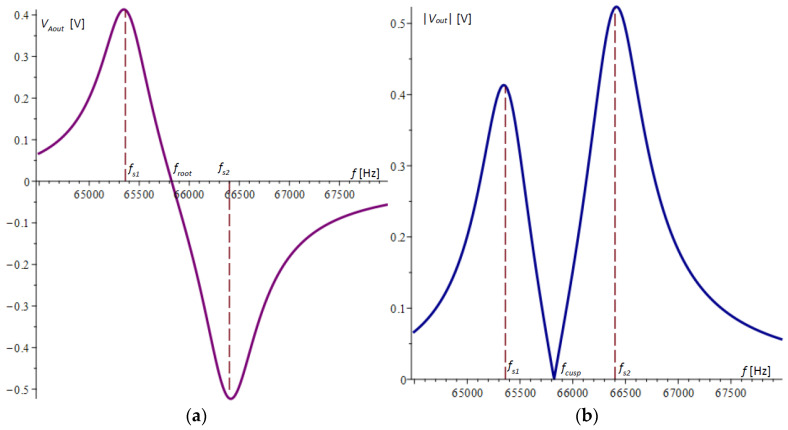
The amplitude–frequency responses obtained by the voltage differences from the half-bridges of the two microcantilevers: (**a**) the difference of the amplitudes of the two microcantilevers; (**b**) the absolute value of the difference of the amplitudes of the two microcantilevers.

The graph of the difference $V_{Aout}$ according to (34) is plotted in Figure 4a. It is noticeable that the extreme points in the figure do not coincide with the natural frequencies of the microcantilevers.

Devices that measure the amplitude–frequency response, such as those in [30], typically convert the voltage by an absolute value, resulting in the graph shown in Figure 4b. In this graph, the cusp point $f_{cusp}$, which in Figure 4a is the root of the amplitude–frequency response, is clearly visible.

The cusp point $f_{cusp}$ has a signal that is below the noise level. Further theoretical and experimental studies will show that this point is sensitive enough to the variation in the natural frequency of one of the microcantilevers to register a mass change in the order of a few femtograms (10^−15^ g).

## 5. Experimental Study of the Dual-Microcantilever Sensor

**Figure 5:**
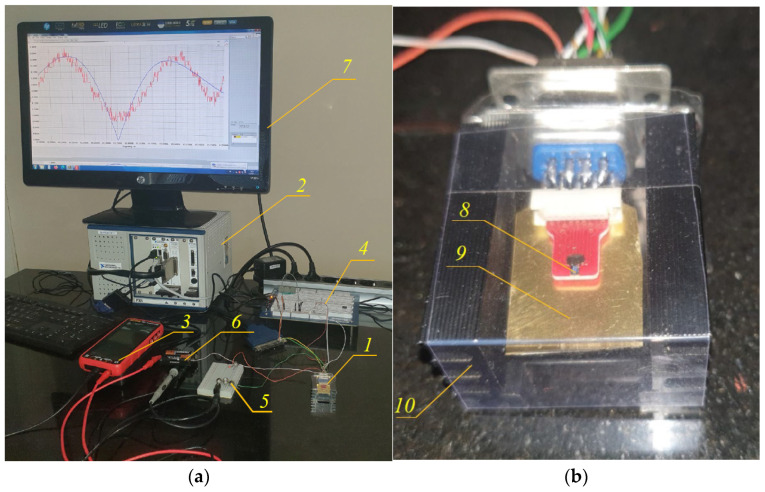
Experimental system for testing piezoresistive sensors with dual-microcantilever beams: (**a**) general view; (**b**) closer look at sensor and its actuation. 1. Sensor. 2. NI PXI system. 3. Ammeter, 4. Digilent wave generator. 5. Potentiometers for adjusting current in microcantilever heaters. 6. Batteries to power heaters. 7. Monitor. 8. Microchip. 9. Piezoelectric actuator. 10. Sensor housing.

In parallel with the development of this theory, an experimental test system was built to verify it. Figure 5a shows the general view of the system. The sensor signals were collected in a National Instruments PXI system with up to 2 MS/s sampling capability. Signal processing was performed using a LabVIEW 11 program capable of measuring high-frequency vibrations with frequencies up to 300 kHz and a resolution of 0.01 Hz [22]. Sensor 1 is elucidated in detail in Figure 5b, where the chip, piezoelectric actuator, and housing can be seen. High-frequency electrical sine signals were produced by a Digilent sine signal generator and fed into a piezoelectric actuator, which excited mechanical vibrations at the base of the two microcantilevers.

Initially, experiments were conducted to verify the theory derived above. For this purpose, vibrations were generated through the sine wave generator, which varied in a range from 64 kHz to 68 kHz, which includes the natural frequencies of the beams. The frequency range was divided into 400 steps, and for each step, vibrations of an order of several periods were generated. For each step, the maximum amplitude between the maximum and minimum voltages was measured for the piezoresistors of the two microcantilevers separately and the output of the Wheatstone bridge. Using the LabVIEW 11 program, the results were saved in an Excel file and then processed in Maple.

**Figure 6:**
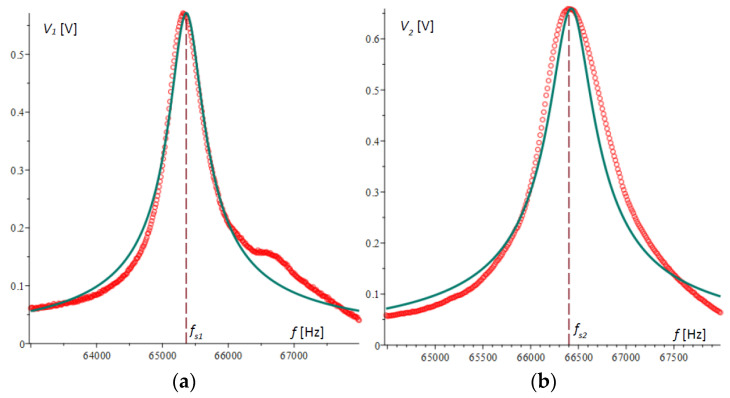
Theoretical and experimental plots of amplitude–frequency response: (**a**) theoretical and experimental amplitude–frequency response of microcantilever 1; (**b**) theoretical and experimental amplitude–frequency response of microcantilever 2.

Figure 6a,b show the plots of the amplitude–frequency responses for microcantilever 1 and microcantilever 2, respectively, obtained experimentally and compared with the theoretical results obtained above. In Figure 6a, the second peak in the experimental plot is not typical and is due to random error.

**Figure 7:**
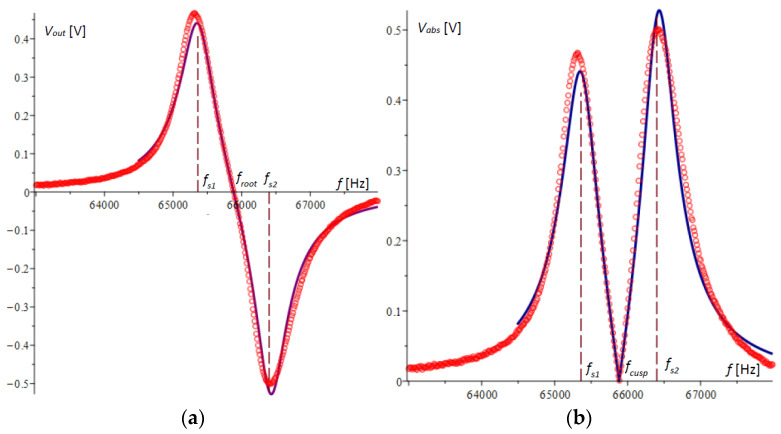
A graphical representation of the amplitude–frequency response results. The experimental results are plotted with a solid line, and theoretical results are represented by a circle symbol: (**a**) the theoretical and experimental amplitude–frequency response of the output voltage of the Wheatstone bridge; (**b**) the theoretical and experimental amplitude–frequency response of the absolute value of the output voltage of the Wheatstone bridge.

Figure 7a shows the voltage differences of the two amplitude–frequency responses obtained by experiment and those from theory, plotted with a solid line. Figure 7b plots the absolute value of the Wheatstone bridge output voltage.

It is evident from the presented graphs that there is a consistent pattern and a reasonably good accuracy of agreement between the theoretical and experimental results. It is noteworthy that in spite of larger deviations in the extreme values, at the cusp point of Figure 7b or the root of Figure 7a, the matching of the two graphs is of higher accuracy. This indicates that the method possesses high robustness, which will be the subject of other studies.

## 6. An Investigation of the Sensitivity of the Method through the Possibilities of Determining the Frequency of the Cusp Point

In Equation (34), the angular frequencies, ω, $\varpi_{1}$, and $\varpi_{2}$ [rad/s], are converted into the rotational frequencies f, $f_{s1}$, and $f_{s2}$ [Hz], respectively, according to the relation

(35) ω=2πf.

For the damping factors $\beta_{1}$ and $\beta_{2}$, the substitution

(36) $$\beta_{i}=2\pi \eta_{i}\;i=1,\;2$$

was used. After ω, $\varpi_{1}$, $\varpi_{2}$, $\beta_{1}$, and $\beta_{2}$ are transformed according to (35) and (36), they are substituted into (34), the numerator of the difference is set to zero, and the equation for the frequency of the cusp point is obtained:

(37) $$\pi^{2}a_{e}^{2}\delta_{r}^{2}\left(\frac{{\tilde{B}}_{2}}{\sqrt{{\tilde{B}}_{2}}}\;-\;\frac{{\tilde{B}}_{1}}{\sqrt{{\tilde{B}}_{1}}}\right)f_{cusp}^{6}+4\left(\frac{{\tilde{B}}_{1}{\tilde{B}}_{2}}{\sqrt{{\tilde{B}}_{1}}}-\frac{{\tilde{B}}_{1}{\tilde{B}}_{2}}{\sqrt{{\tilde{B}}_{2}}}\right)f_{cusp}^{2}=0,$$

where

(38) $$\begin{array}{l} {\tilde{B}}_{1}=\pi^{2}\left[{\left(f_{cusp}^{2}-f_{s1}^{2}\right)}^{2}+4\eta_{1}^{2}f_{cusp}^{2}\right],\\ {\tilde{B}}_{2}=\pi^{2}\left[{\left(f_{cusp}^{2}-f_{s2}^{2}\right)}^{2}+4\eta_{2}^{2}f_{cusp}^{2}\right]. \end{array}$$

The solution of (37) yields an important result for the studies here, by which the cusp point frequency is analytically determined:

(39) $$f_{cusp}=\frac{\sqrt{2}}{2}\sqrt{\frac{f_{s1}^{4}-f_{s2}^{4}}{f_{s1}^{2}-f_{s2}^{2}-2\left(\eta_{1}^{2}-\eta_{2}^{2}\right)}}.$$

By the formula thus derived, it is seen that the dependence of the cusp point frequency on the natural frequencies and damping of the microcantilevers can be analytically investigated.

For the case under consideration and from the data in Table 1, the cusp point in Equation (39) was calculated to be $f_{cusp}$ = 65,889.06309 Hz, which confirms the graphical results for the cusp point in the theory and experiments obtained in Figure 4b and Figure 7b. The same result was confirmed by numerically solving the equation directly composed from (34).

Formula (39) shows that $f_{cusp}$ depends only on the natural frequencies and damping of the system, indicating that the method is stable and is not affected by the amplitude of the excitation base vibrations, the values of the supply voltages, and other system parameters. Since in (39) the squares of the damping factors are subtracted, it is evident that their influence is also small because at close values their difference is zero.

In order to perform an approximate sensitivity analysis of the method, Formula (39) is transformed into the form

(40) $$f_{cusp}=\frac{\sqrt{2}}{2}\sqrt{\frac{f_{s1}^{2}+f_{s2}^{2}}{1-\frac{2\left(\eta_{1}^{2}-\eta_{2}^{2}\right)}{f_{s1}^{2}-f_{s2}^{2}}}}.$$

It is assumed that due to the same geometrical and physical parameters, both beams have almost the same losses, i.e.,

(41) $$\eta_{1}\approx \eta_{2}$$

and Expression (40) can be assumed to be approximately equal to

(42) $$f_{cusp}\approx \frac{\sqrt{2}}{2}f_{s1}\sqrt{1+f_{s21}^{2}},$$

where

(43) $$f_{s21}=\frac{f_{s2}}{f_{s1}}.$$

When the natural frequency $f_{s1}$ of microcantilever 1 increases by a small value $\Delta f_{s1}$, the new cusp point is calculated by the formula

(44) $$f_{cusp1}\approx \frac{\sqrt{2}}{2}\left(f_{s1}+\Delta f_{s1}\right)\sqrt{1+f_{s21}^{2}};$$

where the ratio $f_{s21}$ is assumed to be independent on $\Delta f_{s1}$ due to its small value. Considering (40), the change in the cusp point frequency value in this case is

(45) $$f_{cusp}-f_{cusp1}\approx \frac{\sqrt{2}}{2}\Delta f_{s1}\sqrt{1+f_{s21}^{2}},$$

from which the relationship between the frequency change $\Delta f_{s1}$ and the cusp point change is obtained:

(46) $$\Delta f_{s1}\approx \frac{\sqrt{2}\left(f_{cusp}-f_{cusp1}\right)}{\sqrt{1+f_{s21}^{2}}},$$

considering that the natural frequency of microcantilever 1 can be represented by the expression

(47) $$f_{s1}=\frac{1}{2\pi }\sqrt{\frac{k_{1e}}{m_{1e}}},$$

where $k_{1e}$ and $m_{1e}$ are the effective stiffness and effective mass of cantilever 1, respectively. It follows from (47) that

(48) $$m_{e1}=\frac{k_{1e}}{2\pi^{2}f_{s1}^{2}},$$

Assuming that the effective mass has increased by a small value $\Delta m_{1}$, the new mass is as follows:

(49) $$m_{e1}+\Delta m=m_{e11}=\frac{k_{1e}}{4\pi^{2}f_{s11}^{2}},$$

where $f_{s11}$ is the natural frequency effective variation in microcantilever 1 due to the added mass. By (48) and (49), the difference of the squares of the natural frequencies can be expressed as

(50) $$f_{s1}^{2}-f_{s11}^{2}=\frac{k_{1e}}{4\pi^{2}}\left(\frac{1}{m_{e1}}+\frac{1}{m_{e11}}\right).$$

The resulting expression is transformed after the simplifications

(51) $$f_{s1}^{2}-f_{s11}^{2}=\left(f_{s1}+f_{s11}\right)\left(f_{s1}-f_{s11}\right)\approx 2f_{s1}\left(f_{s1}-f_{s11}\right),$$

are made, because it is assumed that $f_{s1}\approx f_{s11}$ and

(52) $$\frac{1}{m_{e1}}-\frac{1}{m_{e11}}=\frac{m_{e11}-m_{e1}}{m_{e1}m_{e11}}\approx \frac{\Delta m_{e1}}{m_{e1}^{2}}.$$

Here, the simplification is based on the small differences of masses, i.e., $m_{e1}\approx m_{e11}$.

Once the above simplifications are made, the difference in natural frequencies is found:

(53) $$\Delta f_{s1}=f_{s1}-f_{s11}\approx \frac{k_{1e}\Delta m_{e1}}{8\pi^{2}m_{e1}^{2}f_{s1}}=\frac{f_{s1}\Delta m_{e1}}{2m_{e1}}.$$

From (46) and (53), the relationship between the cusp points frequency variation and the effective mass of the microcantilever 1 is given by

(54) $$\Delta m_{e1}\approx \frac{2\sqrt{2}\left(f_{cusp}-f_{cusp1}\right)m_{e1}}{\sqrt{f_{s1}^{2}+f_{s2}^{2}}}.$$

Using this expression, one can calculate what the resolution of the method is; for example, with a measurement limit of $\Delta f_{cusp_{\min}}={\left(f_{cusp}-f_{cusp1}\right)}_{\min}$ = 0.01 Hz, an effective mass of $m_{e1}=\frac{33m_{1}}{280}$ =4.43951 × 10^−11^ kg [23], and beam natural frequencies according to Table 1, a limiting sensitivity lower than 1.5 × 10^−17^ kg or 15 fkg is obtained. Here, the mass of the microcantilever is 14.108 × 10^−10^ kg. From Equation (54), it is concluded that to increase the limiting sensitivity, it is necessary to improve the measurement accuracy and increase the natural frequencies of the microcantilevers. At natural frequencies of the microcantilevers twice as high, for the considered case, the limit resolution is increased by one order of magnitude.

## 7. Experimental Determination of the Capabilities of the Method, Changing One of the Natural Frequencies of the Microcantilevers by Heating

**Figure 8:**
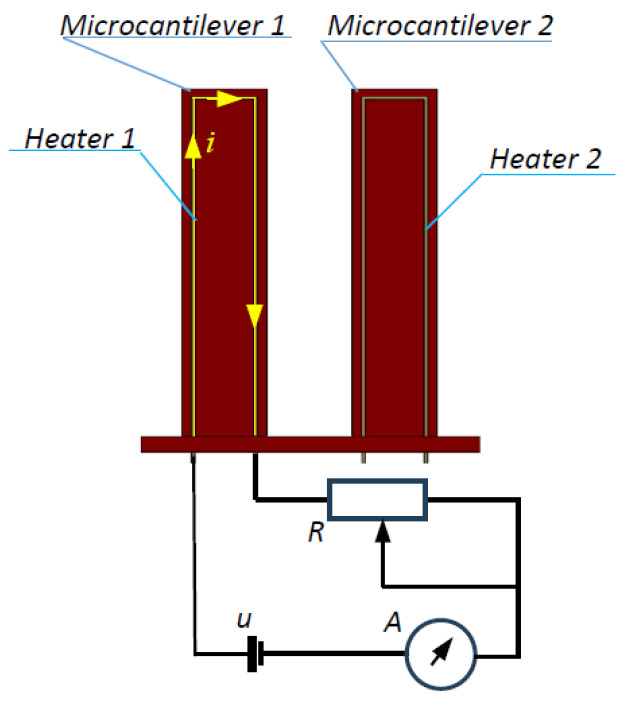
A schematic of the experiment to change the temperature of microcantilever 1 by Joule heating.

The experimental setup shown schematically in Figure 8 was used to investigate the sensitivity of the detection method. The heater of microcantilever 1 was connected to a battery via a serially connected ammeter and a variable resistor. The current in the heater was continuously adjusted through the adjustable resistor, which caused the microcantilever to heat up at different temperatures. As a result of the increased temperature, the microcantilever changed its dimensions in proportion to the coefficient of thermal expansion (CTE), and the natural frequency changed in proportion to a parameter called the temperature coefficient of frequency (TCF) [31,32,33].

The experiments were conducted after the common base of the two microcantilevers was vibrationally driven by setting 400 uniformly varying values in the range of the two natural frequencies of the microcantilevers. The vibration time of each of these frequencies was selected to be greater than five oscillation periods. For each step of these 400 frequencies, the electrical voltages of the two half-bridges were measured and processed according to the methodology described above. The electric current in the heater of microcantilever 1 was varied from 0 to 1800 µA by setting 20 different values. Twenty Technical Data Management (TDM) files [34] were generated using LabVIEW 11 with the recorded values of the differences of the voltage amplitude–frequency characteristics of the two half-bridges. The TDM files were converted to an Excel file and then processed using the Maple program. The Maple program algorithm finds the smallest value of the absolute voltage $V_{abs}$ array and divides it into left $V_{absleft}$ and right $V_{absright}$, and then approximates them with the parabolas, respectively,

(55) $$\begin{array}{l} {\tilde{V}}_{absleft}=a_{l}+b_{l}f+c_{l}f^{2}\\ {\tilde{V}}_{absright}=a_{r}+b_{r}f+c_{r}f^{2} \end{array}.$$

**Figure 9:**
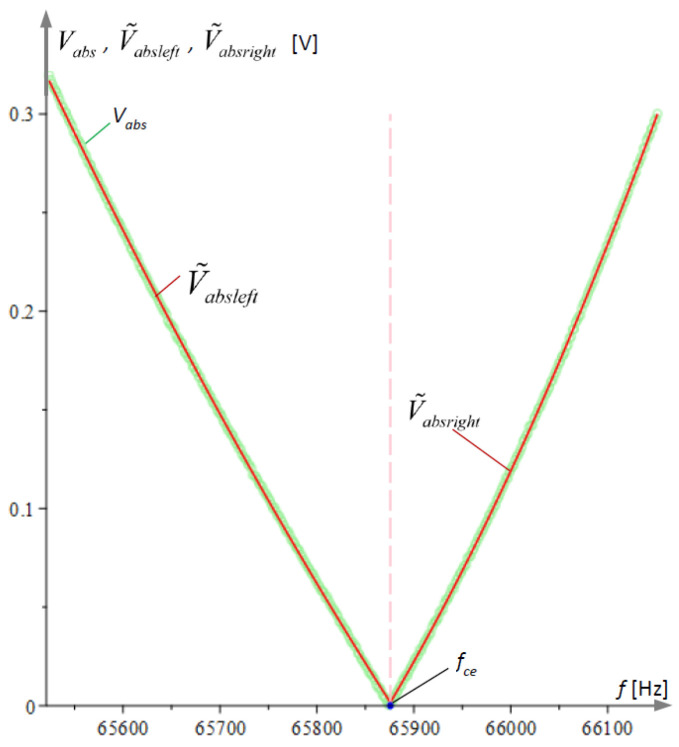
Experimental data processing for $V_{abs}$ from an Excel file obtained at current i = 1053 µA. The frequency of the forced vibrations at the base of the two microcantilevers was varied in the range [65,520, 66,150] Hz.

The intersection $f_{ce}$ of these parabolas is called the experimental cusp point. The processing of the experimental results for a single file is illustrated graphically in Figure 9.

**Figure 10:**
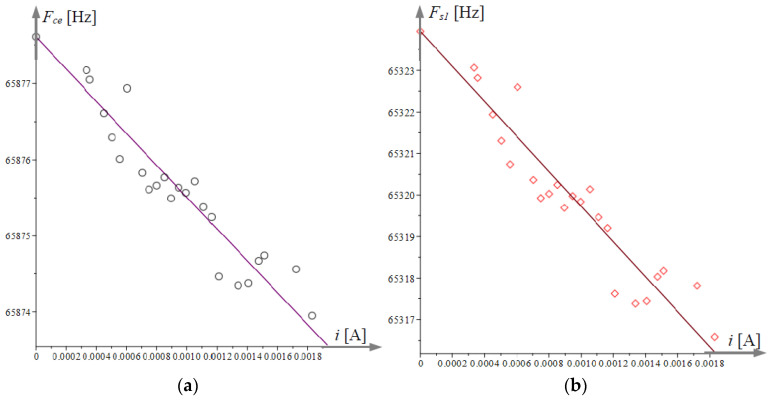
Experimental data on the effect of heating microcantilever 1 on the frequency of the experimental cusp point, natural frequency, and their approximation: (**a**) the cusp point frequency as a function of the heater current and its approximating linear relationship; (**b**) tge dependence of the natural frequency of microcantilever 1 on its heating current *i* and the approximating line.

Figure 10a illustrates the obtained experimental relationship between the cusp point frequencies $f_{ce}$ and the values of the heating current *i*. The experimental data are approximated linearly by a line of the type $f_{capr}\approx bi+c$, depicted in Figure 10a with a solid line. The natural frequency of microcantilever 1 is expressed by (39) and the results with their corresponding linear approximation are plotted in Figure 10b.

## 8. Determine the Sensitivity of the Detection Method by Examining the Offset of the Cusp Point

In order to investigate the sensitivity of the sensor with respect to the mass added to microcantilever 1, we assume here the addition of a thought uniform homogeneous layer of mass $\Delta m_{scv}$ of the same density as that of the beam material to the active surface of the microcantilever. Then, the thickness $l_{3}$ of the beam will increase by $\Delta l_{3}$ as the added mass $\Delta m_{scv}$ is calculated by the formula

(56) $$\Delta m_{SCV}=\rho l_{1}l_{2}\Delta l_{3},$$

where

(57) $$\Delta l_{3}=\frac{\Delta m_{SCV}}{\rho l_{1}l_{2}}.$$

The natural frequency of microcantilever 1 with the additional layer according to [35,36,37,38] is calculated by

(58) $$f_{1SCV}={\frac{1.875104069}{2\pi }}^{2}\sqrt{\frac{EI_{SCV}}{\rho A_{SCV}l_{1}^{4}}},$$

whereby replacing

(59) $$I_{SCV}=\frac{l_{2}{\left(l_{3}+\Delta l_{3}\right)}^{3}}{12},$$

(60) $$A_{SCV}=l_{2}\left(l_{3}+\Delta l_{3}\right),$$

and taking (57) into account, after transformations, the following is obtained:

(61) $$f_{1SCV}=0.161540067953243\frac{m+\Delta m_{scv}}{l_{1}^{3}l_{2}\rho }\sqrt{\frac{E}{\rho }},$$

where $m=l_{1}l_{2}l_{3}\rho$ is the mass of microcantilever 1 without the added thought layer. Here, the calculations are performed with double precision to reflect small masses on the order of femtograms.

If the above formula is substituted with Δm=0, one arrives at the simplification

(62) $$f_{s1}=0.1615400679053\frac{l_{3}}{l_{1}^{2}}\sqrt{\frac{E}{\rho }},$$

which is the natural frequency of the unchanged microcantilever 1.

In order to investigate the correlation between the added mass and the frequency, the frequency difference was found:

(63) $$\Delta f_{1}=f_{1SCV}-f_{s1}=1.609414378345883929\times 10^{14}\Delta m_{scv},$$

from which the inverse dependence follows

(64) $$\Delta m_{scv}=6.213440201943362337853559\times 10^{-15}\Delta f_{1}.$$

**Figure 11:**
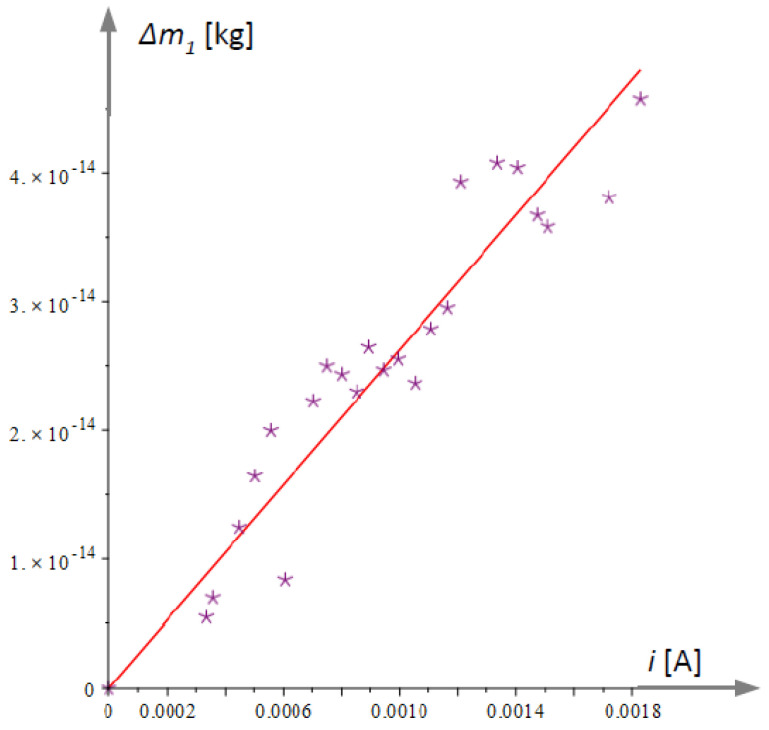
Added thought mass that causes the same variation in the natural frequency of microcantilever 1 as that caused by current heating.

Figure 11 shows the equivalent change in mass that would have resulted from the temperature change of the natural frequency of microcantilever 1, illustrated by Figure 10b. In the figure, the processed experimental data are represented by an asterisk symbol, and the approximated dependence according to Equation (64) is plotted as a solid straight line.

By the coefficients in front, $\Delta m_{scv}$ and $\Delta f_{1}$ are the frequency sensitivities from the mass and vice versa, respectively. By (63) and (64), it is calculated that when the experimental system can be measured to the nearest 0.1 [Hz], it will register a mass that, for every hundredth of a Hz, increases by 6.21344 × 10^−16^ kg. This corresponds to 6.21344 × 10^5^ fg. The mass of a SARS-CoV-2 virus is on the order of 1 fg = 1 × 10^−18^ kg. One person was found to carry 10^10^ to 10^11^ viruses with a total mass of 1–100 µg during the peak of infection [39,40].

The result obtained here indicates that with the accuracy of the measurement system and the adopted parameters of the microcantilevers thus established, it will be possible to detect SARS-CoV-2 viruses at the initial stage of infection before infection occurs. The sensitivity of the system allows it to be applied to the detection of other viruses, pathogens, and chemical substances. There is some margin for improvement in sensitivity in the accuracy of the system itself. Increasing the natural frequency of the microchannels will also have a beneficial effect on sensitivity. This study showed that of the two microcantilevers, the one with the higher natural frequency has a higher sensitivity, and hence, it is advisable to keep this one active.

## 9. Conclusions

The theoretical foundations of a new method for the detection of objects with masses on the order of femtograms are described and justified. Although sensors with two microcantilevers have a well-known structure with one active and another passive microcantilever, and their investigation methods are well known, a new method is proposed here that allows us to sum the amplitude–frequency responses of the voltages at the two Wheatstone half-bridges, thus avoiding the influence of the phase shift of the signals from the piezoresistive sensors in the microcantilevers. Another novelty is the measurement of the cusp point frequency of the amplitude–frequency response of the differences of the two half-bridge voltages, which provides higher accuracy compared to offset-based eigenfrequency methods.

A formula was derived that gives the relationship between the cusp point frequency and the natural frequencies of the microcantilevers. The relationship between the cusp points frequency variation and the effective mass of microcantilever 1 was obtained. The analytical relationships for the variation in the mass of microcantilever 1 and the cusp point frequency were derived, which also determine the sensitivity of the method.

A high-precision experimental system was designed to investigate the method, by which controlled harmonic excitation was generated at the common base of the microcantilevers, and simultaneously, the voltages obtained from piezoresistive sensors formed on the microcantilevers were measured. Using a LabVIEW 11 program in real time, the excitation of the base was simultaneously controlled, and the result obtained due to the deformations of the piezoresistive sensors was measured. The experimental system combined with the proposed measurement method allowed the measurement of the cusp point frequency in the amplitude–frequency response with a resolution of 0.01 Hz, which is sufficient to detect the presence of SARS-CoV-2 virus at an early stage before infection has occurred.

The method was verified by a temperature shift of the frequency of microcantilever 1, and the offsets of the natural frequency and the magnitude of the equivalent femtogram mass that would cause it were determined.

The dual-microcantilever piezoresistive sensor with the experimental system described here and the measurement method applied are universal in nature and can find various applications in medical, chemical, environmental, and other research.

## Figures and Tables

**Table 1:** Microcantilever geometry and materials data.

Parameter	Symbol	Value	Unit
Length of microcantilever 1	$l_{11}$	294 × 10^−6^	m
Length of microcantilever 2	$l_{12}$	292 × 10^−6^	m
Width of microcantilever 1	$l_{21}$	150 × 10^−6^	m
Width of microcantilever 2	$l_{22}$	172 × 10^−6^	m
Height of microcantilever 1	$l_{31}$	4 × 10^−6^	m
Height of microcantilever 2	$l_{32}$	4 × 10^−6^	m
Basis resistance of a piezoresistor	$R_{0}$	1000	Ω
Density of the silicon	ρ	2329 *	kg/m^3^
Young’s modulus of the n-silicon in [110] direction	$E_{110}$	170 *	GPa
Piezoresistivity coefficient for direction 11 n-Si	$\pi_{11}$	−102 × 10^−11^ **	Pa^−1^
Piezoresistivity coefficient for direction 12 n-Si	$\pi_{12}$	53 × 10^−11^ **	Pa^−1^
Piezoresistivity coefficient for direction 44 n-Si	$\pi_{44}$	−14 × 10^−11^ **	Pa^−1^
Stiffness for n-type silicon plane 100 in axis [110]	$C_{11}$	165.65 × 10^9^ ***	Pa
Stiffness for n-type silicon plane 100 in axis [010]	$C_{12}$	63.94 × 10^9^ ***	Pa
Stiffness for n-type silicon plane 100 in axis [001]	$C_{44}$	79.51 × 10^9^ ***	Pa
Natural circular frequency of microcantilever 1	$\varpi_{1}$	10,402.535	s^−1^
Natural circular frequency of microcantilever 2	$\varpi_{2}$	10,568.028	s^−1^
Natural frequency of microcantilever 1	$f_{s1}$	65,361.057	Hz
Natural frequency of microcantilever 2	$f_{s2}$	66,400.888	Hz
Supplying voltage	$v_{cc}$	8	V
Effective amplitude of the external vibrations	$a_{e}$	9.92 × 10^−8^	m
Damping factor of microcantilever 1	$\beta_{1}$	1554.755	s^−1^
Damping factor of microcantilever 2	$\beta_{2}$	1675.886	s^−1^

* According to data from [26]. ** According to data from [27]. *** According to data from [28].

## Data Availability

The data are contained within the article.

## References

[1] Yen Y.-K., Lai Y.-C., Hong W.-T., Pheanpanitporn Y., Chen C.-S., Huang L.-S. (2013). Electrical Detection of C-Reactive Protein Using a Single Free-Standing, Thermally Controlled Piezoresistive Microcantilever for Highly Reproducible and Accurate Measurements. Sensors.

[2] Hansen K.M., Ji H.-F., Wu G., Datar R., Cote R., Majumdar A., Thundat T. (2001). Cantilever-based optical deflection assay for discrimination of DNA single-nucleotide mismatches. Anal. Chem..

[3] Tian X., Tao J., Xu M., Lin Y., Zhao J. (2024). Design and Simulation of an Ultra-Low-Power Hydrogen Sulfide Gas Sensor with a Cantilever Structure. Micromachines.

[4] Chen Z., Cai G., Li Y., Chen Q., Wu W. (2019). Performances and improvement of coupled dual-microcantilevers in sensitivity. Sens. Actuators A Phys..

[5] Tang L., Xu P., Li M., Yu H., Li X. (2020). H2S gas sensor based on integrated resonant dual-microcantilevers with high sensitivity and identification capability. Chin. Chem. Lett..

[6] Xin W., He Z., Zhao C. (2023). Design and Experimental Evaluation of a Dual-Cantilever Piezoelectric Film Sensor with a Broadband Response and High Sensitivity. Micromachines.

[7] Agarwal D.K., Nandwana V., Henrich S.E., Josyula V.P.V.N., Thaxton C.S., Qi C., Simons L.S., Hultquist J.F., Ozer E.A., Shekhawat G.S. (2022). Highly sensitive and ultra-rapid antigen-based detection of SARS-CoV-2 using nanomechanical sensor platform. Biosens. Bioelectron..

[8] Hawari H.F., Wahab Y., Azmi M.T., Shakaff A.Y., Hashim U., Johari S. Design and Analysis of Various Microcantilever Shapes for MEMS Based Sensing. Proceedings of the 2014 International Conference on Science & Engineering in Mathematics, Chemistry and Physics (ScieTech 2014).

[9] Wideband F.T. (2009). low-noise optical beam deflection sensor with photothermal excitation for liquid-environment atomic force microscopy. Rev. Sci. Instrum..

[10] Pedrak R., Ivanov T., Ivanova K., Gotszalk T., Abedinov T., Rangelow I.W., Edinger E., Tomerov E. (2003). Micromachined atomic force microscopy sensor with integrated piezoresistive sensor and thermal bimorph actuator for high-speed tapping-mode atomic force microscopy phase-imaging in higher eigenmodes. J. Vac. Sci. Technol. B.

[11] Jani N., Chakraborty G. (2021). Parametric Resonance in Cantilever Beam with Feedback-Induced Base Excitation. J. Vib. Eng. Technol..

[12] Wang X.D., Li N., Wang T., Liu M.W., Wang L.D. (2007). Dynamic characteristic testing for MEMS micro-devices with base excitation. Meas. Sci. Technol..

[13] Zhou J., Li P., Zhang S., Huang Y., Yang P., Bao M., Ruan G. (2003). Self-excited piezoelectric microcantilever for gas detection. Microelectron. Eng..

[14] Napoli M., Bamieh B., Turner K. (2004). A Capacitive Microcantilever: Modelling, Validation, and Estimation Using Current Measurements. J. Dyn. Syst. Meas. Control.

[15] Zhao R., Boudou T., Wang W.-G., Chen C.S., Reich D.H. (2013). Decoupling Cell and Matrix Mechanics in Engineered Microtissues Using Magnetically Actuated Microcantilevers. Adv. Mater..

[16] Zhang H., Yang S., Zeng J., Li X., Chuai R. (2023). A Genosensor Based on the Modification of a Microcantilever: A Review. Micromachines.

[17] To C.W.S. (1982). Vibration of a Cantilever Beam with a Base Exitation and Tip Mass. J. Sound Vib..

[18] Repetto C.E., Roatta A., Welti R.J. (2012). Forced vibrations of a cantilever beam. Eur. J. Phys..

[19] Lai W.P., Fang W. (2001). A novel antistiction method using harmonic excitation on the microstructure. J. Vac. Sci. Technology. A Vac. Surf. Film..

[20] Lobontiu N., Garcia E. (2004). Two Microcantilever Designs: Lumped-Parameter Model for Static and Modal Analysis. J. Microelectromechanical Syst..

[21] Stavrov V., Stavreva G., Tomerov E. (2020). Tester for Detection of Infectious Agents in Fluid. Bulgarian Patent.

[22] Banchelli L.F., Ganev B.T., Todorov T.S. Sustainability Validation of a LabVIEW Based System for Biomarkers Detection. Proceedings of the XXXII International Scientific Conference Electronics—ET2023.

[23] (2024). Theoretical Background of SPM, 2.1.2 Deflections under the Vertical (Normal) Force Component, NT-MDT Spectrum Instruments. https://www.ntmdt-si.com/resources/spm-theory/theoretical-background-of-spm/2-scanning-force-microscopy-.

[24] Liu C. (2011). Foundations of MEMS.

[25] Allen J.J. (2005). Micro Electro Mechanical System Design.

[26] Lindroos V., Tilli M., Lehto A., Motooka T. (2010). Handbook of Silicon Based MEMS Materials and Technologies.

[27] Smith C.S. (1954). Piezoresistance effect in germanium and silicon germanium and silicon. Phys. Rev..

[28] Hall J.J. (1967). Electronic Effects in the Elastic Constants of “n”-Type Silicon. Phys. Rev..

[29] Sarma M.S. (2001). Introduction to Electrical Engineering.

[30] (2024). RedPitaya, “STEMlab 125-14 Low Bare OEM,” Red Pitaya. https://redpitaya.com/product/stemlab-125-14-low-noise-bare-oem/.

[31] Jiang B., Huang S., Zhang J., Su Y. (2021). Analysis of Frequency Drift of Silicon MEMS Resonator with Temperature. Micromachines.

[32] Zhang R.X., Fisher T., Raman A., Sands T.D. (2009). Linear Coefficient of Thermal Expansion of Porous Anodic Alumina Thin Films from Atomic Force Microscopy. Nanoscale Microscale Thermophys. Eng..

[33] Silva M.A.S., Fernandes T.S., Sombra S. (2012). An alternative method for the measurement of the microwave temperature coefficient of resonant frequency (τf). J. Appl. Phys..

[34] National Instruments Corp (2024). “NI TDMS File Format—What Is a TDMS File?” NI. https://www.ni.com/en/support/documentation/supplemental/06/the-ni-tdms-file-format.html.

[35] Meirovitch L. (1986). Elements of Vibration Analysis.

[36] Rao S.S. (2007). Vibration of Continuous Systems.

[37] Voltera E., Zachmanoglou E.C., Charles E. (1965). Dynamics of Vibrations.

[38] Whitney S. (1999). Vibrations of Cantilever Beams: Deflection, Frequency, and Research Uses. https://emweb.unl.edu/Mechanics-Pages/Scott-Whitney/325hweb/Beams.htm.

[39] Sender R., Bar-On Y.M., Gleizer S., Bernsthein B., Flamholz A., Phillips R., Milo R. (2021). The total number and mass of SARS-CoV-2 virions. Proc. Natl. Acad. Sci. USA.

[40] Ym B.-O., Flamholz A., Phillips R. (2020). SARS-CoV-2 (COVID-19) by the numbers. Elife.

